# Analysis of the temporal trend for consequences of occupational accidents in Brazil (2008 to 2017) 

**Published:** 2019-08

**Authors:** Everaldo Correia de Lima Júnior, Vincius da Silva Oliveira, Érika Carvalho de Aquino, Nelson Azevedo Barros

**Affiliations:** ^*a*^Universidade Fernando Pessoa, Brazil.; ^*b*^Universidade Federal de Goiás, Brazil.

## Abstract

**Background:**

Accidents at work are those that occur in the exercise or in the course for the work activity. They can lead to death or injury, which may lead to temporary or permanent reduction of the capacity to work. In the world, there are approximately 2.3 million deaths annually and 317 million work-related accidents. Reducing these data will lead to a large decrease in the risk to human health. In low and middle income countries, occupational accidents are responsible for 18% of deaths, while in high-income countries, 5%. In this way, the objective of the present study is to analyze the temporal trend of the consequences of occupational accidents in Brazil from 2008 to 2017, in order to evaluate the impact of safety on people s lives and on government.

**Methods:**

The numerical data of the accidents were obtained from the Statistical Yearbook of Accidents of Work. Population data were available on the platform of the Brazilian Institute of Geography and Statistics. The incidence for each State of the typical, path, occupational diseases, with work accident communication, “without work accident communication” and total (liquidated) accidents in the study. Trend analysis was performed using Prais Winsten s linear regression model. The average rates of annual increase were calculated. Stata 14.0 was used for the statistical analysis of the data.

**Results:**

There was a predominance of a decreasing trend. However, in the States of the North and Northeast Regions there was a steady or even growing tendency for several indicators. There was a decreasing trend in Brazil as a whole.

**Conclusions:**

It is suggested that the predominance of reduction tendencies is related to the reduction of the Brazilian GDP. However, future public policies must address the problems observed mainly in the North and Northeast regions of the country.

**Keywords:**

Safety, Trend, Accident, Analysis

